# Supplementary material for: Ultrafast photoinduced C-H bond formation from two small inorganic molecules
Source: Nat Commun. 2024 Apr 2;15:2854. doi: 10.1038/s41467-024-47137-3 (PMC10987588; doi:10.1038/s41467-024-47137-3)
Supplement: Supplementary file 1 — Supplementary Information [file 41467_2024_47137_MOESM1_ESM.pdf]

Supplementary Information for

Ultrafast photoinduced C-H bond formation from two small inorganic molecules

Zhejun Jiang[1], Hao Huang[1], Chenxu Lu[1], Lianrong Zhou[1], Shengzhe Pan[1], Junjie Qiang[1], Menghang Shi[1], Zhengjun Ye[1], Peifen Lu[1], Hongcheng Ni[1,2,*], Wenbin Zhang[1,†], and Jian Wu[1,2,3,4,‡]

[1]*State Key Laboratory of Precision Spectroscopy, East China Normal University, Shanghai 200241, China*

[2]*Collaborative Innovation Center of Extreme Optics, Shanxi University, Taiyuan, Shanxi 030006, China*

[3]*Chongqing Key Laboratory of Precision Optics, Chongqing Institute of East China Normal University, Chongqing 401121, China*

[4]*CAS Center for Excellence in Ultra-intense Laser Science, Shanghai 201800, China*

[*] hcni@lps.ecnu.edu.cn

[†] wbzhang@lps.ecnu.edu.cn

[‡] jwu@phy.ecnu.edu.cn

Table of Contents

Supplementary Note

1. Experimental setup for the optical layout. 3
2. The fitting parameters. 3
3. Identification of different fragmentation channels. 4
4. Evolution of nuclear distance in simulations. 6
5. Roaming mechanism. 6
6. Other initial configurations. 8
Supplementary References 10

Supplementary Note 1. Experimental setup for the optical layout.

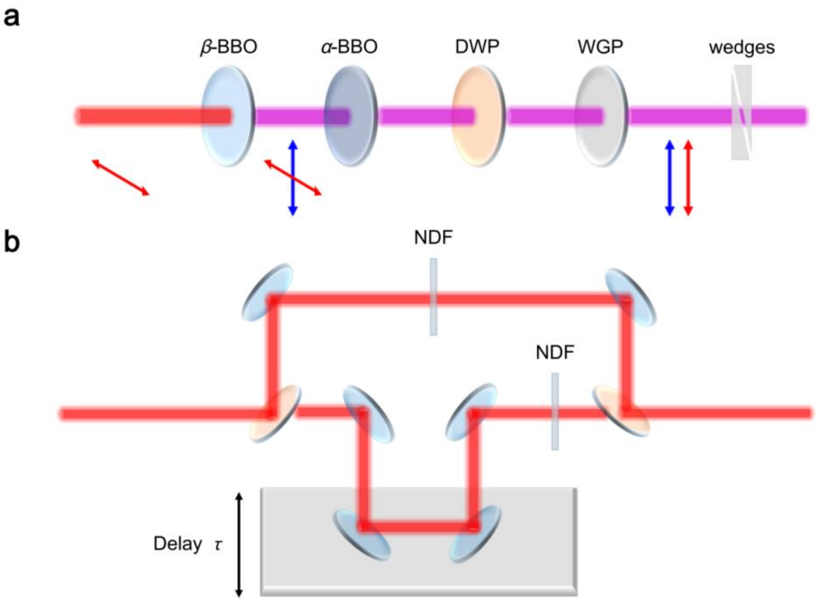

Supplementary Fig 1. Schematic diagram of the setups for two-color and pump-probe schemes. **a** Scheme of collinear generation of two-color laser pulse. DWP, dual-wavelength wave plate; WGP, wire grid polarizer. **b** Experimental pump-probe setup. NDF, neutral density filters.

Supplementary Note 2. The fitting parameters.

In Figs. 2c-2f of the main text, the distributions of the phase-dependent asymmetry amplitude are fitted using sinusoidal function $A(\phi_L) = A_0\sin[\pi(\phi_L\text{-}\phi_{offset})]$, where A_0 and ϕ_{offset} are the amplitude and phase offset of the asymmetry parameter, respectively. The fitting parameters for Figs. 2c-2f are extracted as following: (1) Fig. 2c for C^+ emission in (C^+, O^+) channel: $A_0 = 0.28 \pm 0.002$, $\phi_{offset} = 0.48 \pm 0.002$; (2) Fig. 2d for the HCO^+ emission in (HCO^+, H^+) channel: $A_0 = 0.12 \pm 0.008$, $\phi_{offset} = 0.49 \pm 0.022$; (3) Fig. 2e for H^+ emission in (H^+, H) channel: $A_0 = 0.10 \pm 0.001$, $\phi_{offset} = 0.8 \pm 0.004$; (2) Fig. 2f: $A_0 = 0.08 \pm 0.011$, $\phi_{offset} = 1.50 \pm 0.041$.

In Fig. 3b of the main text, the kinetic energy release (KER) integrated yield distributions of (HCO^+, H^+) channel is fitted by using an exponential function $Y(\mathrm{t}) = Y_0 + \alpha_0\exp(\text{-t}/\tau)$, where α_0 and Y_0 are the amplitude and offset of the (H^+, HCO^+) yield, and

τ is the time constant. In the fitting, the parameters α_0 = -379.0 ± 15.0, Y_0 = 381.0 ± 6.3, τ = 198 ± 16.7 are used.

In Fig. 4d of the main text, the histogram shows the formation time distributions of (HCO^+, H^+) channel extracted in the molecular dynamics simulations. The histogram is fitted by using the Gamma distribution $f(t)=(t^{k-1}e^{-t/\delta})/(\Gamma(k)\tau^k)$, where k=3 is the shape parameter, δ is the scale parameter, and $\Gamma(k)$ is the gamma function $\int_0^{+\infty} y^{k-1}e^{-y}dy$. In the fitting, the used parameters δ= 100.89 fs.

Supplementary Note 3. Identification of different fragmentation channels.

In the data analysis, the involved two-body Coulomb-exploded double ionization channels are unambiguously identified by using the photoion-photoion coincidence (PIPICO) spectrum. As shown in Fig. 1b in the main text, the signals of ion pair originating from the two-body breakup of the same molecular entity are constrained along the sharp diagonal lines of the time-of-flight correlation map. Several two-body fragmentation channels can be identified. The (H^+, H^+) and (C^+, O^+) channels are produced from the Coulomb-exploded double ionization of H_2 and CO monomers, respectively. The direct Coulomb explosion of H_2-H_2, CO-CO, H_2-CO dimer induced by two-site double ionization contributes to the (H_2^+, H_2^+), (CO^+, CO^+), and (H_2^+, CO^+) channel, respectively. Besides these ion pairs, the (H^+, HCO^+) with mass-to-charge ratio (m/q) of ion pair being m/q = 1 and 29 is also identified, which originate from the laser-induced molecular reactions of the H_2-CO dimer. Note that the false coincidence fragmentation events and background ionization events are also recorded in the PIPICO spectrum. For instance, the vertical and horizontal sharp lines in the PIPICO spectrum with m/q = 1, 2 and 28 are the ion fragments of H^+, H_2^+, and CO^+ from the false coincidence fragmentation events. The recoil momentum rule of $p_{\mathrm{sum}} = |\mathbf{p}_{\mathbf{ion1}} + \mathbf{p}_{\mathbf{ion2}}| <$ 4 a.u. is imposed for the sum-momentum of coincidently detected two ion fragments to suppress the false coincidence signal. The discrimination of the detected H^+ ion signals from the dissociative single ionization channel (H, H^+) and double ionization channel (H^+, H^+) relies on the different H^+ ion recoil momentum in the two channels. Typically,

the recoil momentum of H^+ ion from the (H, H^+) channel as accessed via bond softening mechanism is below 15 a.u., which is relatively lower than that from the (H^+, H^+) channel (>20 a.u.). Therefore, a momentum gate of $p_{H^+} = \sqrt{\mathbf{p}_{\mathbf{ionx}}^2 + \mathbf{p}_{\mathbf{iony}}^2 + \mathbf{p}_{\mathbf{ionz}}^2} < 15$ a.u. is employed for the momentum distribution of H^+ to clearly distinguish the H^+ ion signal in (H, H^+) channel from that in (H^+, H^+) channel, where $\mathbf{p}_{\mathbf{ionx}}$, $\mathbf{p}_{\mathbf{iony}}$, $\mathbf{p}_{\mathbf{ionz}}$ are the ion momentum distributions along x, y, and z direction, respectively.

For the investigated (H^+, HCO^+) channel, the fast and slow pathways can be clearly distinguished in the raw data because the ion fragments with high and low KER during the fragmentation will be registered on the detector with different time-of-flight (TOF) or impact position X/Y information. If the laser polarization is along z direction, i.e., the TOF direction, the slow and fast pathway can be clearly distinguished in the PIPICO spectrum, as featured at the central part (slow pathway) and shoulder part (fast pathway) of the sharp TOF correlation diagonal line. While when the laser polarization is along y direction, as shown in Fig. 1c of the main text, the signals of ion fragments with same mass from slow and fast pathway are overlapped in the TOF axis, which thus cannot be distinguished in the PIPICO spectrum. In such case, however, since the ion fragments from the two pathways have different emission momentum along y direction, as shown in Supplementary Fig. 1, the slow and fast pathway of (H^+, HCO^+) channels can be easily distinguished when considering both the position Y and TOF information.

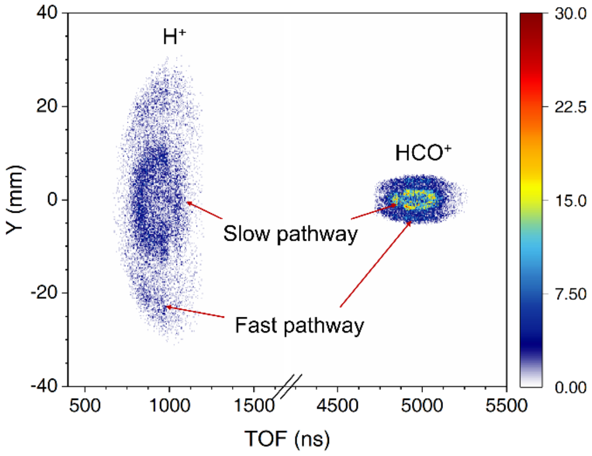

Supplementary Figure 2. Measured position Y-TOF spectrum of the ion fragments produced in (H^+, HCO^+) channel. The slow (inner ring) and fast (outer ring) pathway are indicated by red arrows.

Supplementary Note 4. Evolution of nuclear distance in simulations.

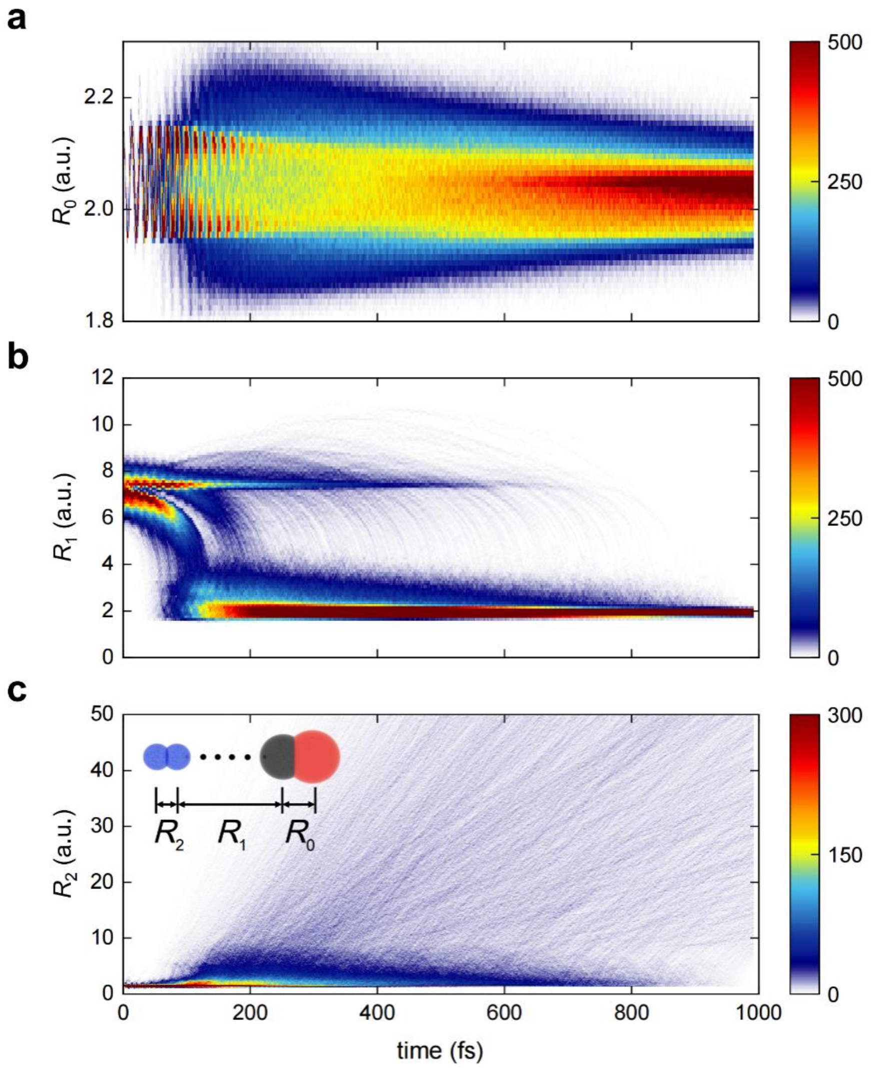

Supplementary Figure 3. Evolution of nuclear distance in the molecular dynamics simulations. **a-c** Simulated evolution of nuclear coordinates **a** R_0, **b** R_1, **c** R_2 in a $H_2 \cdot\cdot CO$ dimer, which eventually leads to the formation of HCO^+. The definition of the reaction coordinates (R_0, R_1, and R_2) is depicted in the inset in **c**.

Supplementary Note 5. Roaming mechanism.

We have shown in the main text that HCO^+ cannot be formed if H_2 is initially placed at the O side in a colinear configuration. Here, we further show that the scenario of the roaming of H_2 from the O side to the C side and subsequent formation of HCO^+ is also negligible.

To address such scenario, we performed molecular dynamics simulations. We have evolved one thousand trajectories on the potential energy surface (PES) of cationic $(H_2\text{-}CO)^+$ in Jacobi coordinates (θ, R_1, R_2) shown in Supplementary Fig. 3 with the initial orientation angle $\theta = -\pi$ and the initial position (R_1, R_2) sampled by the ground-state nuclear wave function for the PES ($\theta = -\pi$, R_1, R_2). The time evolution of the trajectories in Supplementary Figs. 3(a)-(b) shows that in most cases the two molecules have moved away from each other within 200 fs, as their intermolecular distance R_1 increases with time and the H-H bond length R_2 remains stable without breaking. Supplementary Fig. 3(c) shows that the change of the orientation angle θ of most trajectories is less than 0.1π over 200 fs, which means that H_2 molecule have moved away from CO molecule well before it roams from O side to C side. Therefore, the contribution of the H_2 roaming mechanism to the formation of HCO^+ is negligible.

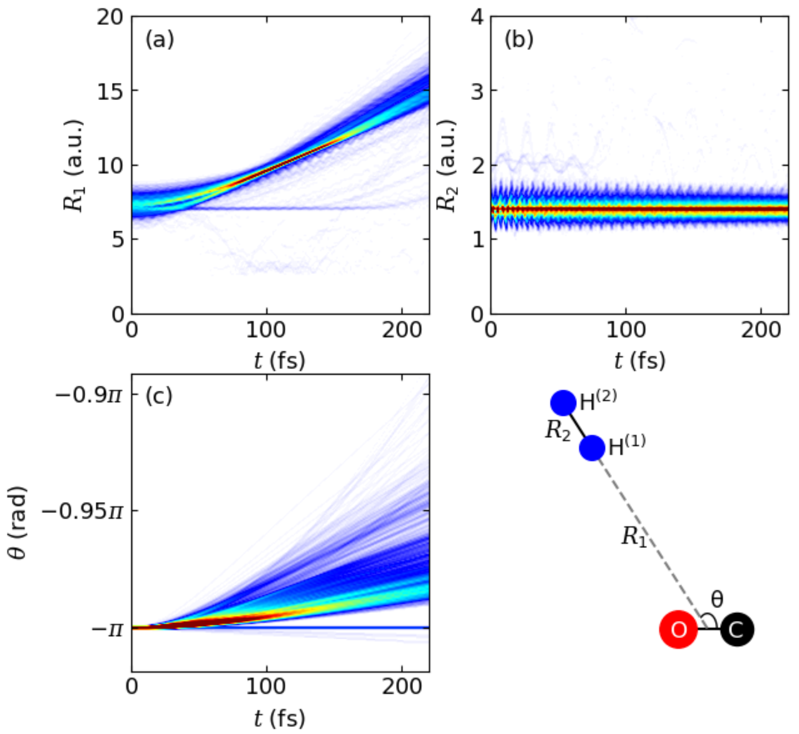

Supplementary Figure 4. Simulated evolution of nuclear coordinates (a) R_1, (b) R_2, (c) θ in the $(H_2\text{-}CO)^+$ dimer. The distance of the C-O bond is fixed at 1.1565 Å, R_1 is the vector pointing from the midpoint of the C-O bond to $H^{(1)}$, R_2 is the vector pointing from $H^{(1)}$ to $H^{(2)}$, which always keeps the same direction as R_1 in this configuration. θ is the angle between the R_1 vector and the C-O bond.

Supplementary Note 6. Other initial configurations.

The potential energies of the neutral H_2-CO dimer in different configurations have been calculated in ref. [1]. As shown in Supplementary Fig. 4(a), the geometry of H_2-CO can be described naturally using the Jacobi coordinates (R, θ_1, θ_2, ϕ,r_{CO},r_{H2}). There, R is a vector pointing from the centre of mass of CO to the centre of mass of H_2, θ_1 is the angle between R and a vector pointing from atom O to C, θ_2 is the angle between R and a vector pointing from $H^{(2)}$ atom to $H^{(1)}$, ϕ the dihedral angle between the two planes defined by vector of R with the CO molecule and with the H_2 molecule, r_{CO} is the bond length of the CO molecule and r_{H2} is the bond length of the H_2 molecule.

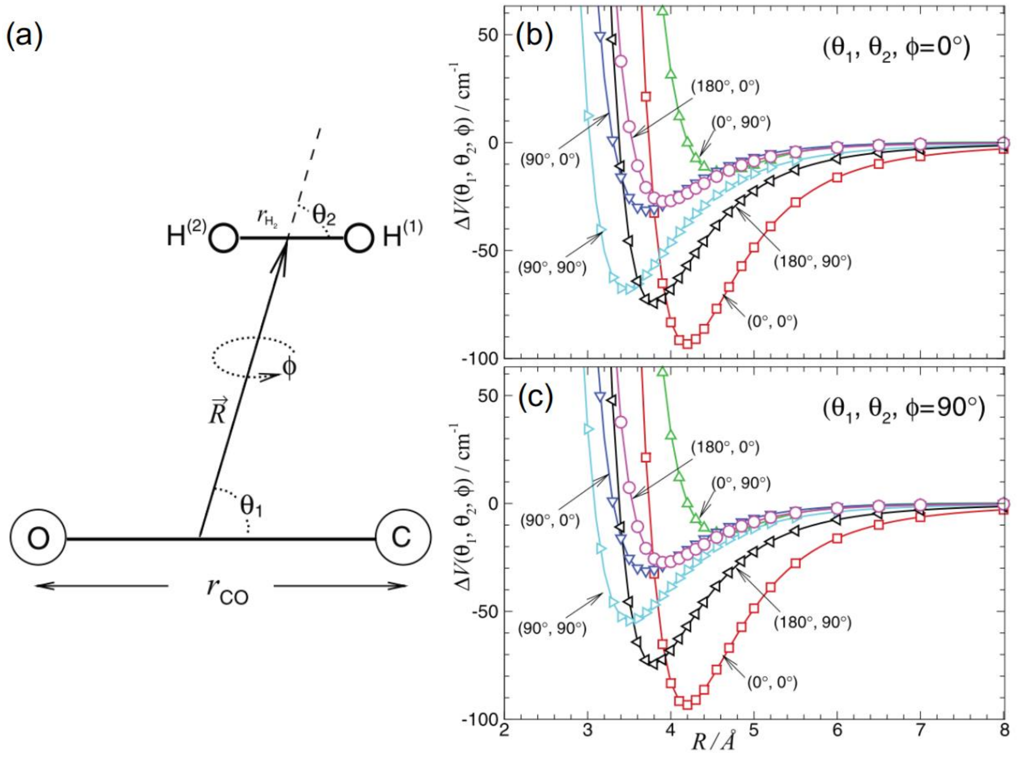

Supplementary Figure 5. (a) Complete Jacobi coordinates for H_2-CO. (b) Vibrational averaged interaction energy curves for neutral H_2-CO at various relative orientations. Figure adapted from Supplementary ref. [1], with the permission of AIP Publishing.

The potential energy for H_2-CO at different relative orientations can be expressed as $\Delta V(\theta_1, \theta_2, \phi)$, as shown in Supplementary Figs. 4(b) and 4(c). Of these curves, the red curve (θ_1=0°, θ_2=0°, ϕ=0°/90°) corresponding to the colinear configuration has much lower energies than other curves, meaning that most of the H_2-CO dimer would exist colinearly with H_2 at the C side. Furthermore, the H_2-CO dimer in other configurations with different relative orientations need to undergo relative intermolecular rotation after single ionization to form HCO^+, which would make the HCO^+ yield much lower than the colinear configuration.

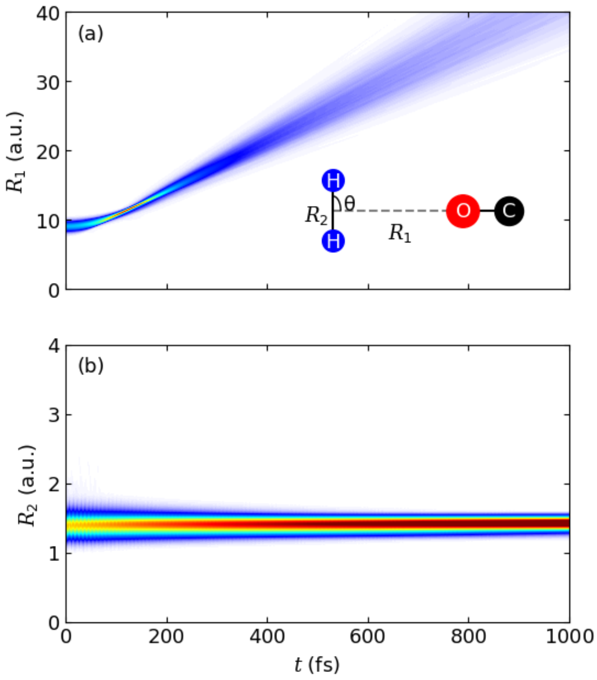

Supplementary Figure 6. Simulated evolution of nuclear coordinates (a) R_1, (b) R_2 in a $(H_2\text{-}CO)^+$ dimer for a vertical configuration with H_2 at O side initially. R_1 is the vector pointing from C atom to the center of mass of H_2, which is always colinear with the C-O bond orientation in this configuration. R_2 is the nuclear distance of H_2 molecule. θ is the relative orientation of H_2 and CO molecules.

Except the most stable initial colinear configuration as indicated by the red curve in Supplementary Fig. 4, we have performed molecular dynamics simulations starting additionally from the second most stable vertical configuration with H_2 at the O side corresponding to the black curve (θ_1=180°, θ_2=90°, ϕ=0°/90°) in Supplementary Figs. 4(b) and 4(c). In such case, the rotation and stretching of the H-H bond are needed for H_2 to react with CO to form HCO^+. In the simulations, we set up the Jacobi coordinates (θ, R_1, R_2) as defined in the inset of Supplementary Fig. 5(a). We subsequently evolved 10000 trajectories with the initial orientation angle θ = 90° and the initial position sampled by the ground-state nuclear wave function for PES (θ = 90°, R_1, R_2). Supplementary Figs. 5(a) and 5(b) show the evolution of all trajectories in R_1 and R_2 coordinates over time. Most trajectories reflect that the two molecules are moving away from each other with the absolute value of R_1 increasing and R_2 remaining stable, meaning that little HCO^+ can be formed in this situation.

Therefore, we draw the conclusion that the colinear configuration with H_2 at C side is the main configuration for the formation of HCO^+ from H_2 and CO, for its high population due to the lowest potential energy and the high yield owing to the formation process without a reaction barrier or any necessity of relative rotation.

Supplementary References

[1] Hui Li, Xiao-Long Zhang, Robert J. Le Roy, Pierre-Nicholas Roy, "Analytic Morse/long-range potential energy surfaces and predicted infrared spectra for CO–H_2 dimer and frequency shifts of CO in (para-H_2)$_N$ N = 1–20 clusters", *J. Chem. Phys.* **139**, 164315 (2013).
